# Mechanosensing and Regulation of Cardiac Function

**DOI:** 10.4172/2155-9880.1000314

**Published:** 2014-06-05

**Authors:** David E Dostal, Hao Feng, Damir Nizamutdinov, Honey B Golden, Syeda H Afroze, Joseph D Dostal, John C Jacob, Donald M Foster, Carl Tong, Shannon Glaser, FNU Gerilechaogetu

**Affiliations:** 1 Central Texas Veterans Health Care System, Temple, Texas, USA; 2 Division of Molecular Cardiology, Cardiovascular Research Institute, Texas A&M University Health Science Centre, College of Medicine, Temple, Texas, USA; 3 Systems Biology and Translational Medicine, the Texas A&M University Health Science Centre, College of Medicine, Temple, Texas, USA; 4 Scott & White Healthcare - Digestive Disease Research Centre, Temple, Texas, USA

**Keywords:** Mechanosensing, β1-integrin, AT1 receptor, Cardiac function, Signalling

## Abstract

The role of mechanical force as an important regulator of structure and function of mammalian cells, tissues, and organs has recently been recognized. However, mechanical overload is a pathogenesis or comorbidity existing in a variety of heart diseases, such as hypertension, aortic regurgitation and myocardial infarction. Physical stimuli sensed by cells are transmitted through intracellular signal transduction pathways resulting in altered physiological responses or pathological conditions. Emerging evidence from experimental studies indicate that β1-integrin and the angiotensin II type I (AT1) receptor play critical roles as mechanosensors in the regulation of heart contraction, growth and leading to heart failure. Integrin link the extracellular matrix and the intracellular cytoskeleton to initiate the mechanical signalling, whereas, the AT1 receptor could be activated by mechanical stress through an angiotensin-II-independent mechanism. Recent studies show that both Integrin and AT1 receptor and their downstream signalling factors including MAPKs, AKT, FAK, ILK and GTPase regulate heart function in cardiac myocytes. In this review we describe the role of mechanical sensors residing within the plasma membrane, mechanical sensor induced downstream signalling factors and its potential roles in cardiac contraction and growth.

## Introduction

Heart failure is the leading cause of morbidity and mortality in developed countries. Cardiac dysfunction in patients with hypertension-induced heart failure is characterized by reduced left systolic and diastolic ventricular function, which is associated with myocyte hypertrophy and ventricular re-modeling. Although the pathophysiological mechanisms associated with pressure overload-induced cardiac hypertrophy have the focus of intense scientific investigation for over 3 decades, the cellular mechanisms remain poorly understood [1,2]. There is abundant evidence that regulation of protein phosphorylation through intracellular kinases and phosphatases is a key mechanism by which cells respond to extracellular stimuli [2-8]. In this area of research, using in vivo and in vitro models, both β1-integrin and the angiotensin II type I (AT1) receptor have been shown to serve as mechanosensors, which can temporally regulate contractile function in cardiac myocytes [3,8-15]. Since mechanical sensors and their downstream signalling factors have an important roles in the regulation of contractile function and diastolic function may provide a new therapeutic approach for the treatment of diastolic heart disease.

## Mechanical Sensors

A growing body of evidence indicates that extracellular binding proteins and G-protein coupled receptors and associated signalling pathways play critical roles in sensing and transducing mechanical stress into biochemical signals that coordinate cardiac contraction and play major roles in the pathological progression of cardiac disease. In this mini review we discuss two major mechanical sensors, Integrins and AT1 receptors.

## Integrins

Mechanical load induced hypertrophic growth of the adult heart is caused by signals beginning at the cell surface through receptors and integrins play a very important mechanical sensors in cardiac myocytes [16]. Integrins are a family of cell-surface receptors that link the extracellular matrix (ECM) to the cellular cytoskeleton at places called focal adhesion sites [17-19]. Integrins are heterodimeric molecules comprised of non-covalently associated α and β subunits. A given α-subunit may interact with more than one β-subunit, resulting in 24 different heterodimers identified to date. Cardiac myocytes express a limited set of integrin subunits, which include α1, α3, α4, α6, α7, α10 and α11, β1, β3 and β5 [20-24]. The specificity of integrin signalling is made possible by α and β-subunits that form the heterodimeric pair. The α-subunit generally confers ECM specificity [17,25], whereas the β-subunit interacts with the cytoplasmic environment. Ligand binding to the extracellular integrin domain induces conformational changes and integrin clustering for activation of signalling cascades and recruitment of multiprotein complexes to focal adhesions [26,27]. Because integrins lack enzymatic activity, activation of signalling factors requires interaction with cellular proteins that have kinase activity. In non-cardiac cells, the cytoplasmic tail of the β-subunit has been shown to directly bind to several cytoskeletal proteins that associate with signalling molecules [28]. In cultured neonatal rat cardiac myocytes, β1 integrin has been shown to be important for coupling mechanical stretch to activation of MAPKs, as well as focal adhesion kinase (FAK) and Rho GTPases [29-31]. Angiotensin II (Ang II) and other growth factors stimulate cardiac myocyte contraction and adhesion via β1 and αvβ3 integrins, which involve inside-to-outside signalling mechanisms [20,22-24]. Ang II also orchestrates adhesion through upregulation of various integrins (αv, β1, β3, β5), as well as expression of cytoskeletal protein, such as α-actinin, which is intimately connected to integrins at the site of focal adhesions [24]. However, the role of integrins in the regulation of cardiac myocyte contraction remains to be systematically studied under both physiologic and pathologic conditions. In non-cardiac tissues, physiological stretch has been shown to regulate contractility primarily through integrins that couple to FAK activation [32]. It is therefore possible that FAK coupled integrins, such as β1 integrin could also regulate contractile force in cardiac myocytes. This would imply that integrins could serve as novel targets for the therapy in patients with contractile dysfunction.

## Angiotensin II Type 1 Receptors (AT1R)

Mechanical stress is the most important stimulus for the development of cardiac hypertrophy. Actually, mechanical stress induces a variety of hypertrophic responses in cardiac myocytes [33]. Furthermore, pretreatment of cardiac myocytes with AT1 receptor blockers (ARBs) significantly attenuates all of these mechanical stretch-induced hypertrophic responses [34,35]. AT1 receptor is a well-known seven transmembrane-spanning G protein coupled receptor (GPCR) that has significant contribution for the development of cardiac hypertrophy [36]. Early studies revealed the involvement of autocrine/paracrine mechanisms through stretch-induced release of AngII. Recent studies show that the AT1 receptor can be activated by mechanical stress through an Ang II-independent mechanism [36,37]. It is well recognized that AT1 receptor is the first mechanosensitive GPCR component that mediates transformation of mechanical stimuli into biochemical information and gives rise to mechanosensor induced different cellular responses (such as inflammation, cell growth, and differentiation etc.) [36,38]. Inverse agonists, such as candesartan, which stabilizes the AT1 receptor in an inactive conformation, suppresses AT1 activation by both mechanical stress and Ang II [39]. Mechanical stretch induced activation of the AT1 receptor produces an anticlockwise rotation and a shift of transmembrane (TM) 7 into the ligand binding pocket [39]. Recent studies suggest that mechanical stretch induces β-arrestin-biased signalling downstream of AT1 receptors in the absence of ligand or G protein activation [40]. Mechanical stretch triggered an AT1 receptors mediated conformational change in β-arrestin similar to that induced by a β-arrestin-biased ligand to selectively stimulate receptor signalling in the absence of detectable G protein activation [40]. Yatabe et al., demonstrated that mechanical stress caused an increase in the phosphorylation levels of ERK in rat mesangial cells (RMCs) through the Ang II independent AT1 receptor activation [37]. An angiotensin receptor blocker (ARB), olmesartan, was found to attenuate ERK activation induced by mechanical stress. Several studies have reported that under mechanical stretch the concentrations of secreted Ang II and the levels of angiotensinogen expression were unchanged [41,42]. Although AT1 has been shown to couple to signalling pathways that regulate intracellular calcium, a potential role of AT1 in mediating stretch-induced changes in cardiac myocyte contractility remain to be explored. A deeper understanding of the cellular and molecular mechanisms responsible for activation and regulation of AT1 mediated signalling may help identify new pharmacologic agents that can be used to regulate cardiac contractile function and hypertrophy.

## Signalling Factors

Mechanical sensors can be activated by mechanical stretch leading to activation of multiple classic signalling pathways involving in alterations of a large number of signalling molecules, e.g. focal adhesion kinase, Rho family GTPases, Integrin-linked kinases, MAP kinases and AKT. These activated multiple signalling pathways respectively use their own classic signalling pathways to regulate heart functions.

**Figure 1:**
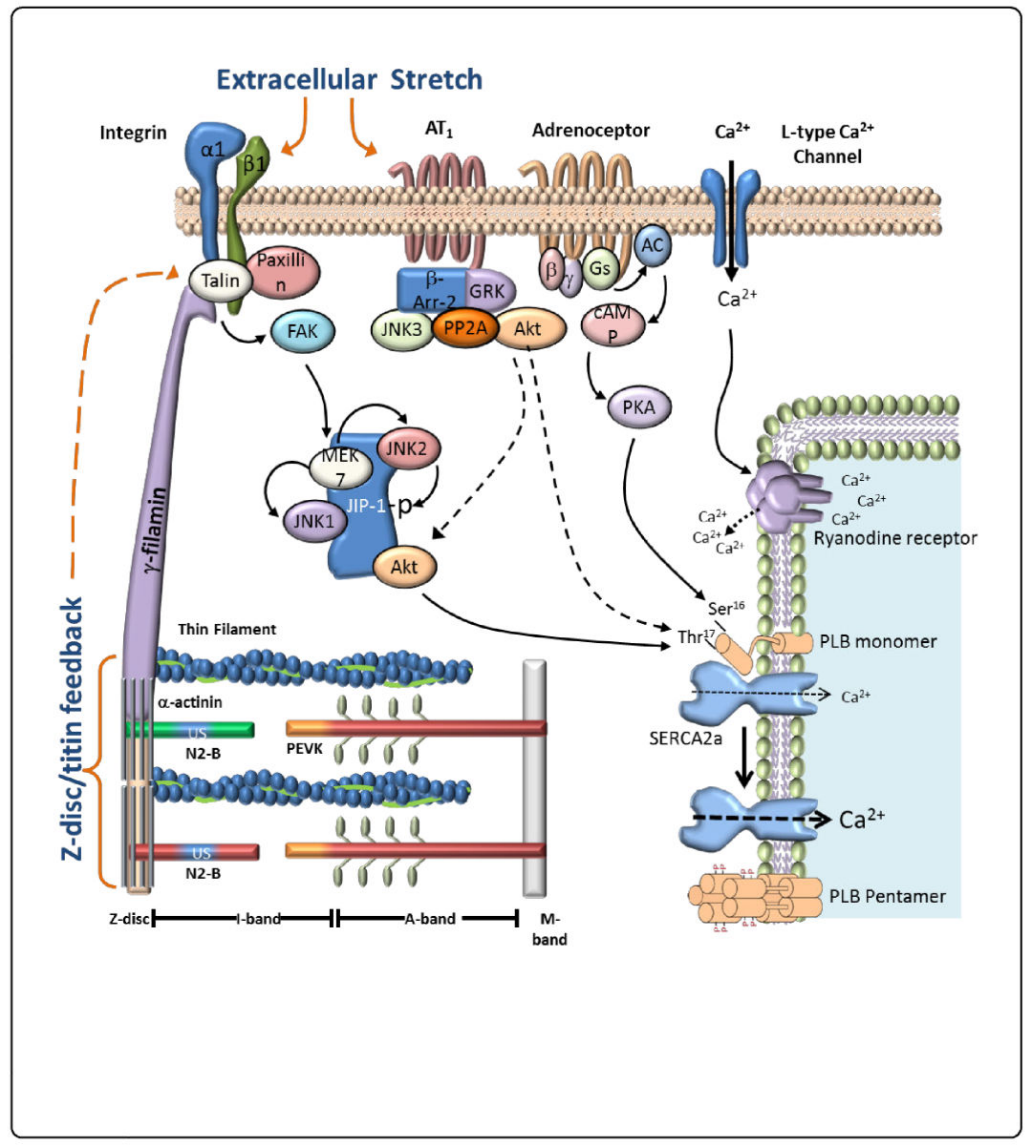
Schematic of regulation of cardiac myocytes function by mechanical stretch.

Mechanical stretch can lead to activation angiotensin II type 1 receptor and integrins. Activation of these proteins can initiate several downstream signalling pathways, such as MAPK and AKT, which can alter contractile function by leading to changes in intracellular calcium ion concentration. Abbreviations: AT1R, Angiotensin II Type 1 Receptor; ERK, extracellular signal regulated kinase; JNK, c-jun N-terminal kinase; p38, p38 mitogen activated protein kinase; FAK, focal adhesion kinase; PP2A, protein phosphatase-2 A; RYR, ryanodine receptors; SR, sarcoplasmic reticulum; SERCA, sarcoplasmic reticulum calcium-ATPase (Figure 1).

## Focal Adhesion Kinase (FAK)

Focal adhesion kinase (FAK) is a tyrosine-phosphorylated protein that localizes to integrin-enriched cell adhesion sites [43,44]. FAK directly binds to the cytoplasmic tail of β-integrin and thereby plays a major role in integrin-mediated signalling [45]. Although FAK is an essential kinase, as indicated by the fact that null mice are embryonically lethal; the function of FAK in the heart has been controversial. Several groups advocate the cardioprotective nature of FAK while others disagree [46-49]. A number of exciting new animal models have now clearly established a role for FAK in the development of the cardiovascular system and possibly in heart disease. At the cellular level, FAK controls cell migration, proliferation and survival [46,50]. FAK is involved in proliferation processes and extracellular mechanical signalling in the heart, and is highly expressed in the myocardium. Recent studies indicate that FAK is important for transducing mechanical stimuli in isolated cardiac myocytes, fibroblasts and in mechanically overloaded myocardium [29,46]. Transgenic mice with cardiac myocyte overexpression of cardiac myocyte FAK demonstrate concentric cardiac hypertrophy, suggesting that FAK selectively regulates signalling mechanisms that govern myocyte growth in width, which could be important for the adaptive response to increases in cardiac afterload [51]. In cardiac myocytes, mechanical stretch induces FAK phosphorylation at Tyr397, Tyr861 and Tyr925, which yet remains to be shown to play a role in cardiac myocytes contractile function [29]. The temporal dynamics, molecular interactions and abilities of FAK to sense contractile force and transduce mechanical stretch are basic questions which remain to be resolved for cardiac cells under physiologic and pathologic conditions.

## Rho family GTPases

The Ras homologous (Rho) family of small GTPases control a large number of cardiac functions in the heart. Dysregulation of these small G proteins has been demonstrated to have pathological consequences in the cardiovascular system. Mechanical stretch activates the Rho GTPases, Rac1 and RhoA, which participate in focal adhesion formation and activation of growth pathways. Integrins are involved in the regulation of the activities of several members of the Rho family of small GTPases, which control the growth or contraction of filamentous actin fibers and myosin [52]. Several tyrosine kinase members, such as the Src family, are also involved in the transduction of signals from integrin to Rho GTPases. Previously it has been reported that Src, either alone or in association with other classes of tyrosine kinases, has the ability to regulate the Rho GTPase activation cycle by modulating guanine-exchange factor and GTPase activating proteins [53]. In addition, experiments utilizing cardiac fibroblasts isolated from neonatal rat hearts treated with dominant-negative Rac1 or RhoA adenoviruses and subjected to mechanical stretch, revealed an activating role for Rac1 and an inhibitory role for RhoA in FAK activation that resulted in AKT473 phosphorylation [31]. In contrast to Rac1, previous studies suggest that RhoA is a mediator of hypertrophic responses in the myocardium [54,55]. Inhibition of the RhoA affecter ROCK, using the ROCK inhibitor GSK 576371, prevented left ventricular hypertrophy and reduced collagen deposition, which were accompanied by improved diastolic function in pressure overload-induced cardiac hypertrophy in the rat [56]. The effect of Rho GTPase on regulation of mechanical stretch in cardiac myocytes contractility has not been clarified. Emerging evidence indicates that Rho GTPases, contribute to cardiac excitation-contraction coupling mechanisms by controlling intracellular Ca^2+^ signalling and phosphorylation/dephosphorylation. RhoA has been associated with regulation of the L-type Ca^2+^channel and regulation of SERCA2 expression in cardiac myocytes [57,58]. There is also evidence that Rac1, together with Pak1 may regulate contractility by reduce cytosolic Ca^2+^ mobilization by altering L-type Ca^2+^ channels and/or ryanodine gates via dephosphorylation by protein phosphatase 2A [59,60]. Although these proteins have been identified as potential targets for the development of new therapeutic strategies in the treatment of heart failure, future efforts remain to be performed which will better understand the mechanisms and identify the molecular partners that regulate the activities of Rho GTPases in the heart.

## Integrin-linked kinase (ILK)

ILK is a widely expressed serine/threonine kinase that binds to the C terminus of β1-integrin [61]. ILK links extracellular matrix interactions to cellular processes such as remodeling of cytoskeletal proteins, growth, proliferation, survival, and differentiation [12]. To date, a large number of proteins associated with mechanosensing have been shown to bind to different domains of ILK. It binds to α-actinin via β-parvin/affixin and forms a complex with PINCH and thymosin β4 [12]. It has been shown to phosphorylate myosin light chain, GSK-3β (glycogen synthase kinase-3β), and AKT/PKB [62]. Several genetic loss-of-function studies in flies, worms, and mice have revealed embryonic death due to cell adhesion and cytoskeletal defects [12]. The conditional cardiac knock-out in mice leads to DCM and sudden cardiac death [63]. Bendig et al. applied a forward genetic screen in zebrafish and identified an L308P mutation in the zILK gene causing progressive loss of contractility in zebrafish hearts [14]. This mutation disrupted the interaction with β-parvin/affixin, suggesting that its presence is essential for normal cardiac function and potentially cardiac stress sensing [14]. Likewise, in another zebrafish study, a nonsense mutation (Y319X) led to a dysmorphic ventricle with reduced cardiac function combined with severe endothelial defects, similar to alterations observed in mice lacking the integrin-binding extracellular matrix protein laminin α4 [64]. Cardiac-restricted overexpression of ILK induces cardiac hypertrophy via activation of ERK and p38 MAPK, hence suggesting ILK to be a proximal prohypertrophic signalling activator [13]. Little is known regarding the role of ILK in cardiac myocyte contraction. The localization of ILK localization to costameres and z-discs suggests that ILK plays a crucial role in the ability of the heart to adapt to changing workloads. The exact roles of ILK as both a mechanosensor and regulator of myocyte contraction under normal and pathological conditions therefore remain to be elucidated.

## The mitogen-activated protein kinase (MAPK) pathway

Mitogen-activated protein kinases (MAPKs) are serine/threonine kinases that become activated upon tyrosine/threonine phosphorylation and additional modifications, and then in turn phosphorylate and activate nuclear substrates (such as c-myc, c-jun, ATF-2, and p62) and other kinases (such as p90 and MAPKAP kinase) [29,30,65-68]. The three best characterized MAPK cascades are the extracellular-regulated kinases (ERK), the c-Jun N-terminal kinases (JNK) and the p38 MAPKs cascade, the latter two belong to the group of stress-activated protein kinases (SAPKs). Studies from our lab and others indicate that ERK, JNK and p38 are activated by mechanical stretch in isolated neonatal rat ventricular myocytes [29,30]. Although MAP kinases have been shown to participate in the regulation of cardiac contractility, the underlying mechanisms are poorly understood and appear to be different for each of MAPK cascades. Acute p38 activation has been shown to reduce force development and activate protein phosphatase-2A (PP2A) in ventricular myocytes [69]. PP2A activation not only affects calcium handing by dephosphorylating PLB, but is localized to the Z-disc, where it can “re-tune” contractile filaments by dephosphorylating regulatory proteins troponin-I and tropomyosin. Recent studies indicate that the B56α targeting protein of the PP2A complex localizes to the Z-disc, but moves away with α-adrenergic stimulation [70]. Previous studies showing that JNK activation downregulates B56α expression and mRNA stability in cardiac myocytes, provides evidence that JNK can regulate contractility at the myofilament level [71]. Although JNK is well-known to have major roles in transcriptional regulation and apoptosis, its role as a regulator of intracellular Ca^2+^in cardiac myocytes is a novel function which remains to be completely understood.

## Protein kinase B (AKT)

AKT, also referred to as protein kinase B, is a serine/threonine kinase found as part of the insulin, insulin-like growth factor-1 (IGF-1)4/phosphatidylinositol 3-kinase (PI3K)/phosphatidylinositol-dependent kinase-1 (PDK1) pathway [72]. Upon activation, AKT phosphorylates a broad range of substrates involved in metabolism, transcription, translation, cell growth, differentiation, proliferation, and survival [73,74]. In the heart the IGF-1/ AKT axis is implicated in the control of physiological cardiac hypertrophy, contractile function, and Ca^2+^ handling [75-82].

Associations between AKT activity and calcium handling proteins were initially observed in experimental models of cardiomyopathy wherein decreased AKT activation was concurrent with diminished SERCA, NCX, and PLB phosphorylation [83]. Conversely, in transgenic mice with cardiac specific overexpression of AKT, it was shown that the amplitude of Ca^2+^ current was enhanced in AKT myocytes compared with that in wild-type myocytes, which may be at least in part responsible for the enhanced cellular Ca^2+^ transients [76,84]. Second, an increased protein expression of SERCA could be identified as another molecular mechanism in transgenic mice expressing cardiac specific constitutively active AKT. Adenoviral gene transfer of the transgene into rat myocardium [85,86] recapitulates this phenotype. Recently, another study showed that activated AKT phosphorylates PLB at Thr17, providing a new mechanism whereby the preferential translocation of AKT to the SR is responsible for enhancement of contractility without stimulation of hypertrophy [85]. We have also reported that AKT functionally improves diastolic calcium handling through phosphorylation of PLB at Thr17 by anthrax lethal toxin [87].

Similarly, mice created with cardiac-specific expression of nuclear-targeted AKT also showed enhanced contractility and superphysiological ventricular dynamics, but the molecular mechanisms responsible for the increased cardiac performance were related to increased loading of the SR due to increased phosphorylation of phospholamban (Ser16 PLB) [88]. In addition, it was shown that phosphatase PP1, which dephosphorylates PLB and thereby inhibits SERCA, provides an additional pathway for increased contractility.

## Conclusion

In summary, mechanosensing is required for maintaining normal function in the myocardium. External activation of mechanosensors regulates cardiac development and contractile performance, whereas disruption of this signalling mechanism results in mechanical dysregulation, cardiac hypertrophy and heart failure. Although in vivo and in vitro studies have been widely used to describe the effects of mechanical forces on myocyte structure and function, the signalling pathways that convert the mechanical stimuli into biological and pathological responses remain to be fully understood. Although a number of key mechanosensors and downstream signalling factors have been identified, further research is needed to unravel the regulatory determinants under physiological and pathological conditions. These are of great clinical importance because these mechanisms are an important component of the adaptive response to cardiac disease and heart failure. A better understanding of these stress-dependent signalling pathways will be important for developing novel therapeutic strategies to control the progression cardiac hypertrophy and prevent heart failure.
